# Soffritto: a deep learning model for predicting high-resolution replication timing

**DOI:** 10.1093/bioinformatics/btaf231

**Published:** 2025-07-15

**Authors:** Dante Bolzan, Ferhat Ay

**Affiliations:** Center for Autoimmunity and Inflammation, La Jolla Institute for Immunology, La Jolla, CA 92037, United States; Bioinformatics and Systems Biology PhD Program, University of California, San Diego, La Jolla, CA 92093, United States; Center for Autoimmunity and Inflammation, La Jolla Institute for Immunology, La Jolla, CA 92037, United States; Bioinformatics and Systems Biology PhD Program, University of California, San Diego, La Jolla, CA 92093, United States; Department of Pediatrics, University of California, San Diego, La Jolla, CA 92093, United States

## Abstract

**Motivation:**

Replication timing (RT) refers to the order in which DNA loci are replicated during S phase. RT is cell-type specific and implicated in cellular processes including transcription, differentiation, and disease. RT is typically quantified genome-wide using two-fraction assays (e.g. Repli-Seq) which sort cells into early and late S phase fractions followed by DNA sequencing, yielding a ratio as the RT signal. While two-fraction RT data are widely available in multiple cell lines, it is limited in its ability to capture high-resolution RT features. To address this, high-resolution Repli-Seq, which quantifies RT across 16 fractions, was developed, but it is costly and technically challenging with very limited data generated to date.

**Results:**

Here, we developed *Soffritto*, a deep learning model that predicts high-resolution RT data using two-fraction RT data, histone ChIP-seq data, GC content, and gene density as input. Soffritto is composed of a Long Short-Term Memory (LSTM) module and a prediction module. The LSTM module learns long- and short-range interactions between genomic bins, while the prediction module is composed of a fully connected layer that outputs a 16-fraction probability vector for each bin using the LSTM module’s embeddings as input. By performing both within cell line and cross-cell line training and testing for five human and mouse cell lines, we show that Soffritto is able to capture experimental 16-fraction RT signals with high accuracy, and the predicted signals allow detection of high-resolution RT patterns.

**Availability and implementation:**

Soffritto is available at https://github.com/ay-lab/Soffritto.

## 1 Introduction

Replication timing (RT) refers to the process by which cells duplicate DNA during the S phase of the cell cycle. Different regions of the genome replicate at different times in a cell-type-specific RT program. While the precise regulation of the RT program remains to be fully elucidated, its pattern has been found to correlate with chromatin structure, histone marks, lamin association, and transcriptional activity (Marchal *et al.* 2019). The majority of publicly available RT data is produced using two-fraction, or two-stage, assays such as early/late (E/L) Repli-Seq (Hiratani *et al.* 2008, Marchal *et al.* 2018). In these two-fraction assays cells are pulse-labeled with BrdU and sorted into early and late S phase fractions. Labeled DNA from both fractions are sequenced and the log2 ratio of early to late coverage is computed for fixed-size bins (e.g. 10 or 50 kb) genome wide. This log2 ratio represents the average time at which each bin (relative to all other bins) is replicated across a cell population. Two-fraction RT assays are limited in their ability to capture replication at high temporal resolution (Marchal *et al.* 2018). To address this limitation, multifraction Repli-Seq protocols that sort cells into more than two S phase fractions have been developed, enabling each genomic bin to be characterized by a normalized vector where each value represents the percentage of cells that replicated within the corresponding fraction (Hansen *et al.* 2010). A 16-fraction assay, denoted high-resolution Repli-Seq, is currently the highest resolution multifraction assay. High-resolution Repli-Seq enables the identification of finer scale RT features such as initiation zones (IZs) (regions of the genome that replicate earlier than surrounding regions and at a constant rate), breakages (small bumps in RT due to replication origin activity within timing transition regions), and biphasically replicating regions that are either undetected or only partially detected in two-fraction and low-resolution multifraction RT assays (Zhao *et al.* 2020, Klein *et al.* 2021).

Computational methods have been developed to predict the E/L ratio generated from two-fraction assays using either epigenomic or sequence-based features. Replicon, a mechanistic model, simulates RT using DNase I hypersensitivity, a measure of chromatin accessibility as input (Gindin *et al.* 2014). Another approach based on epigenomic features built least absolute shrinkage and selection operator (LASSO) regression models using chromatin binding proteins and histone modifications as input to predict RT in Drosophila (Comoglio and Paro 2014). TIGER is an algorithm that infers RT profiles from whole-genome sequence data obtained from proliferating cell samples by amplifying read depth variation across loci that occur due to differences in RT (Koren *et al.* 2021). Of the models that utilize DNA sequence, CONCERT, a Long Short-Term Memory (LSTM)-based deep learning model, predicts RT by extracting sequence features such as k-mer counts (Yang *et al.* 2022). These methods predict a single value, the average RT, per genomic bin, and to date no existing computational approaches predict high-resolution (i.e. 16-fraction) RT. Developing such a predictive model would further our understanding of how exogenous features contribute to finer resolution RT patterns. Furthermore, high-resolution Repli-Seq is a technically challenging and costly assay, thus a predictive model would enable researchers to predict the 16-fraction profiles in cell lines for which such data is not yet available.

With that motivation, we developed Soffritto, a deep learning model that predicts 16-fraction RT from a combination of epigenomic and sequence-based features. More specifically, Soffritto takes six histone modifications (H3K27ac, H3K27me3, H3K36me3, H3K4me1, H3K4me3, H3K9me3), GC content, gene density, and two-fraction Repli-Seq data as input at 50-kb resolution to generate a 16-fraction probability vector for each genomic bin. We validated Soffritto in five cell lines with available 16-fraction RT data, three of which are human-derived (H1, H9, HCT116) and two of which are from mouse (mESC and mNPC). We found that Soffritto accurately captures 16-fraction RT by first training and testing within cell lines and then performing cross-cell line validation. We used five different metrics for evaluation that prioritize different patterns in the 16-fraction RT signal that are useful for its biological interpretation. We also found that Soffritto’s predicted RT profiles captured temporal features specific to high-resolution assays. Overall, this work paves the way for the prediction of high-resolution RT program in distinct cell types to further elucidate the relationship between fine-scale RT patterns and other biological processes.

## 2 Methods

### 2.1 Data collection and processing

The processed 16-fraction Repli-Seq data for H1, H9, HCT116, mESC, and mNPC were downloaded from GSE137764 (Zhao *et al.* 2020). The data are in the form of a matrix where each column represents a 50-kb bin, and each row corresponds to an S phase fraction. The rows are sorted from the earliest S fraction (S1) to the latest S fraction (S16). The data are at 50-kb resolution because it is the technical limit for 16-fraction Repli-Seq based on a replication fork speed of 1.8 kb/min and the 30-minute duration of BrdU labeling for each fraction (Zhao *et al.* 2020). The entries of the matrix are Gaussian-smoothed log2 ratios of the read counts per kilobase million (RPKM) of the respective S phase fraction to that of a G1 control. Bins with all S phase fraction values of 0 or N/A were removed from downstream analyses. Bins overlapping ENCODE blacklist regions were also removed to ensure reliable feature data (Amemiya *et al.* 2019). We subsequently normalized each bin such that the 16-fraction values sum to 1. We will refer to these as probability vectors even though each “probability” is a proportion of cells that replicated in the corresponding fraction. These vectors were then used as the labels for model training and testing.

We selected six commonly measured histone modifications (H3K27ac, H3K27me3, H3K36me3, H3K4me1, H3K4me3, H3K9me3) for use as features. Histone ChIP-seq data were downloaded from ENCODE (Luo *et al.* 2020) for H1, H9, HCT116, and mESC and from GEO accession GSE96107 for mNPC (Bonev *et al.* 2017). In all cases, the histone modification signal was downloaded in bigWig file format and represented as fold-change over control (input). To construct a feature vector for each histone mark, the genome was first partitioned into 50-kb bins. For each bin, the feature’s value was represented as the mean fold-change over control.

We downloaded 2-fraction Repli-Seq data for the five cell lines from the 4DN data portal (Reiff *et al.* 2022). We generated two-fraction RT feature vectors for each cell line by closely following the 4DN Repli-Seq processing pipeline scripts found on https://github.com/4dn-dcic/repli-seq-pipeline. Read alignment files were first merged across biological replicates for early and late fractions, respectively. The merged alignments were then filtered, sorted, and deduplicated. RPKM were computed for non-overlapping 50 kb bins for early and late fractions, respectively. The base 2 logarithm ratio of early to late RPKM was used as the two-fraction RT feature vector. To avoid division by 0, a small adjustment factor equal to the most frequent difference between consecutive bin RPKM values is added to the early and late RPKMs, respectively. All accession IDs for the two-fraction Repli-Seq data and the histone ChIP-seq data may be found in Supplementary Table S1.

Two sequence-based features were utilized: gene density and GC content. To construct gene density feature vectors, we ran bedtools intersect to assign a count value to each 50-kb bin that corresponds to the number of overlapping genes with at least one base-pair overlap. Gene coordinates were obtained from GENCODE’s gene annotation (hg38 for human and mm10 for mouse) (Frankish *et al.* 2019). The GC content feature vector was obtained by computing the fraction of base pairs that are either G or C for each 50 kb bin using the hg38 or mm10 reference genome. All nine features were concatenated to form an *n* × 9 feature matrix to use as model input where *n* corresponds to the number of genomic bins for which 16-fraction Repli-Seq data was available for a given set of training data.

### 2.2 Soffritto architecture

**Figure 1.**
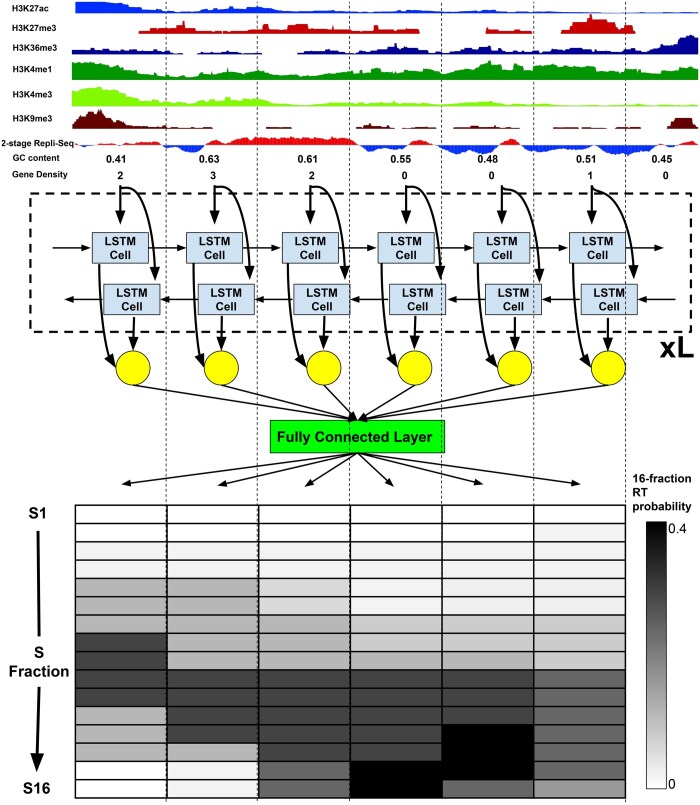
Soffritto architecture. Each vertical dotted line delineates a 50-kb bin. Nine features are fed into a bidirectional, multilayered LSTM module and a prediction module applies a fully connected layer to generate 16-fraction replication probabilities for each bin.

Soffritto consists of an LSTM module and a prediction module (Fig. 1). LSTMs are a variant of recurrent neural networks that are typically applied to sequential data (Hochreiter and Schmidhuber 1997). Each LSTM cell is composed of an input, forget, and output gate, which coordinate how much information is received from the previous time step (genomic bin in this application). For a given genomic bin *t*, batch size *n*, feature dimension *d*, and hidden dimension *h*, the corresponding LSTM cell is represented by the following equations:


$$\begin{array}{c} I_{t}=\sigma (X_{t}W_{xI}+H_{t-1}W_{Ih}+b_{I})\\ \begin{array}{c} F_{t}=\sigma (X_{t}W_{xF}+H_{t-1}W_{Fh}+b_{F})\\ O_{t}=\sigma (X_{t}W_{xO}+H_{t-1}W_{Oh}+b_{O}) \end{array} \end{array},$$

where $I_{t},F_{t},O_{t}\in R^{n\times h}$ represent the input, forget, and output gates, respectively, $X_{t}\in R^{n\times d}$ is the input, $H_{t-1}\in R^{n\times h}$ is the hidden state of the previous time step, $W_{xI},W_{xF},W_{xO}\in R^{d\times h}$ and $W_{Ih},W_{Fh},W_{Oh}\in R^{h\times h}$ are learnable weight parameters, and $b_{I},b_{F},b_{O}\in R^{h\times 1}$ are biases. The outputs of each gate are then combined to update the cell state and the hidden state of each cell as follows:


$$\begin{array}{c} C_{t}=F_{t}⊙C_{t-1}+I_{t}⊙\tanh\left(X_{t}W_{xC}+H_{t-1}W_{Ch}+b_{c}\right)\\ H_{t}=O_{t}⊙\tanh\left(C_{t}\right) \end{array},$$

where $C_{t}\in R^{n\times h}$ represents the cell’s internal state, $W_{xC}\in R^{d\times h}$ and $W_{Ch}\in R^{h\times h}$ are weight parameters, $b_{c}\in R^{h\times 1}$ is a bias vector, and “circle with the dot” represents the Hadamard product of two matrices (i.e. element-wise multiplication). Soffritto’s LSTM module is bidirectional and therefore an identical set of variables and equations is learned in parallel with recursion being performed in the opposite direction, i.e. the genomic bin to the right rather than to the left. Furthermore, Soffritto’s LSTM module is multilayered with a separate set of parameters learned for each layer while depending on the hidden states of the previous layer. Concretely, given L layers and L≥2, input $X_{t}^{l}$ of the lth layer is equal to $H_{t}^{l-1}$, the hidden state of the same bin in the previous layer. In the final layer, each bidirectional $\vec{H_{t}}$,$\overset{\leftarrow }{{\;H}_{t}}$ pair is concatenated to form an n×2h matrix, which is inputted into Soffritto’s prediction module. The prediction module consists of a fully connected layer that generates a matrix of dimension n×16, where each column corresponds to an S phase fraction, sorted from early to late and each row is a genomic bin. Next, a logsoftmax transformation is applied to each row to facilitate training with KL divergence loss. For prediction, an exponential transformation is applied as a final step to ensure that the RT prediction is in probability space for each bin.

### 2.3 Model training

Soffritto was trained using KL divergence loss and L2 regularization to prevent overfitting. The Adam optimization algorithm was used for parameter updates. For intra-cell line training, we left out chromosome 6 for validation and chromosome 9 for testing while the rest of the autosomal chromosomes were used for training. We omitted sex chromosomes to avoid sex bias. For leave-one-cell-line-out training (cross-cell line), we iteratively left out an entire cell line’s data for testing and trained on all autosomes except for chromosomes 6 and 9 from the other four cell lines. The chromosome 6 data from the four cell lines in the training set were used for validation (Supplementary Fig. S1). All models were trained for 100 epochs. A hyperparameter grid search was performed on the validation chromosome by tweaking the number of LSTM layers (1, 2, 3, 4), the batch size (8, 16, 32, 64), the hidden dimension (16, 32, 64, 128, 256), the learning rate (0.00001, 0.0001, 0.001), and the L2 weight decay (0, 0.00001, 0.0001, 0.001). The model with the lowest KL divergence loss on the validation set was selected to predict on the test set. Optimal hyperparameter configurations for intra-cell line and leave-one-cell-line-out training may be found in Supplementary Tables S2 and S3, respectively. Soffritto was implemented in PyTorch (Paszke *et al.* 2019).

### 2.4 Feature-specific baseline predictions

For each feature, we first sorted values from lowest to highest and then partitioned into 16 quantile bins. For each bin we assigned an S fraction label based on its order for both ascending and descending values. For example, in ascending order, the quantile bin with the highest values is given a label of S16, but a label of S1 in descending order. We then assigned S fraction labels to each genomic bin according to its quantile label. For evaluating these predictions, we used mean absolute error between the predicted S fraction labels and the observed Argmax RT fractions across bins.

### 2.5 Baseline predictive models

Given that there are no existing 16-fraction RT prediction methods, we chose the following five models as baselines to contextualize Soffritto’s performance: support vector regression (SVR), linear regression, random forest regression, LASSO linear regression, and Multilayer Perceptron (MLP). All models were implemented using the *scikit-learn* package except for the MLP model, which was trained using Pytorch. For the first three models and for each train-test split, we trained a separate model with default parameters for each S phase fraction. For the LASSO model, we tuned the L1 regularization coefficient using the validation set over the following values: (10^−5^, 10^−4^, 10^−3^, 10^−2^, 10^−1^, 1). The non-neural network-based models’ predicted values were clipped between 0 and 1 and then normalized to sum to one for each genomic bin. The predicted values were clipped prior to normalization to ensure that negative values would not skew the normalization and contradict the probabilistic definition of the labels. The MLP’s architecture is a two-layer neural network with a hidden dimension equal to the best-performing Soffritto model’s hidden dimension for the corresponding train-test split. The MLP was also trained with Adam optimization and KL divergence loss with L2 regularization using the same learning rate and L2 weight decay as Soffritto.

### 2.6 Autocorrelation analysis

Autocorrelation was computed for observed 16-fraction RT chromosome-wise by treating each chromosome as a separate time series, with each 50-kb bin corresponding to a time point. Each fraction was treated as an independent time series, and autocorrelation was computed for lags varying from one to *n*, where *n* is equal to the number of bins in the corresponding chromosome. Then for each lag, autocorrelation values were averaged across all fractions. This yields a single autocorrelation track for a given chromosome. To compute the autocorrelation function for two-stage RT, we utilized the log2 ratio of early to late RPKM for each 50-kb bin as input. We also varied lag from one to *n* in the two-stage RT case. Lastly, we assessed autocorrelation significance by computing 95% and 99% confidence bands for each chromosome under the assumption of a Gaussian distribution *N(0, 1/n)*.

## 3 Results

### 3.1 Soffritto accurately captures high-resolution RT within cell lines

**Figure 2.**
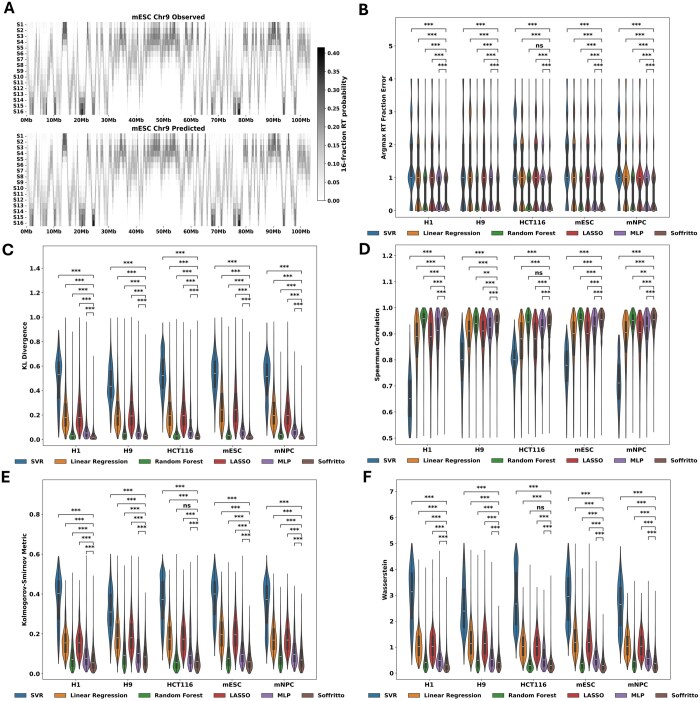
Intra-cell line evaluation of Soffritto on chromosome 9. (A) Observed versus predicted 16-fraction Repli-Seq heatmaps in mESC for the whole chromosome 9. (B–F) Violin plots of the five metrics with each data point corresponding to a bin. All statistical tests were performed using a Wilcoxon signed-rank test between Soffritto’s predictions and each of the baseline models’ predictions. Each Wilcoxon test was one-sided with the alternative hypothesis being that Soffritto’s mean was greater than the baseline model’s mean for Spearman correlation and less than for all other metrics; ns = not significant (*P* > .05), *.01 < *P* < .05, **.001 < *P* ≤ .01, ***P ≤ .001.

To evaluate Soffritto, we trained separate models for each of the five cell lines (H1, H9, HCT116, mESC, and mNPC) on all autosomal chromosomes except for chromosome 6 and chromosome 9, which were used for validation and testing respectively. The baseline models were trained and tested on the same data splits. While Soffritto’s predicted 16-fraction RT heatmaps visually recapitulate experimentally measured data to a great extent (Fig. 2A), we sought to quantitatively assess predictions using five different metrics that capture both the general probabilistic nature of the labels and attributes that are useful for characterizing RT.

#### 3.1.1 Argmax RT Fraction Error

We first examined the S phase fraction which contained the highest replication probability (argmax) for each bin for both observed and predicted RT profiles for chromosome 9. While this Argmax RT fraction does not capture cellular heterogeneity in RT, it provides an overall measure of the dominant fraction at which a genomic bin is replicated. We define the metric Argmax RT Fraction Error (ARFE) as the absolute difference between the Argmax RT fraction of observed and predicted high-resolution RT data. ARFE yields an integer value that lies between 0 and 15 for each bin (argmax fractions are represented as integers from 0 to 15) with 0 being the best possible agreement. ARFE has the advantage of being biologically interpretable: if a bin has an ARFE value of 2, then the model’s prediction is 2 S phase fractions off from the observed/measured data. We found that Soffritto achieves a mean ARFE of less than one for each cell line (e.g. mean of 0.52 for mESC and 0.65 across all five cell lines) as well as a median ARFE of 0 for all cell lines. Compared to the baseline predictive models, Soffritto achieves the lowest mean ARFE significantly in four out of the five cell lines across all bins (Fig. 2B). We also created baseline models for each input feature by partitioning feature values into 16 quantiles and predicting the corresponding S phase fraction based on ascending or descending order (Section 2; Supplementary Fig. S2). We found that Soffritto’s mean ARFE is consistently lower across all cell lines compared to each feature-specific baseline with two-stage RT being the best predictor among all features (mean of 1.21 for mESC and 1.47 across all 5 cell lines; Supplementary Fig. S2).

#### 3.1.2 KL divergence

We next examined how similar the overall predicted 16-fraction probability distribution is to the observed distribution for each bin. To quantify this distance, we computed KL divergence for each bin by setting the observed 16-fraction probabilities as the true probability distribution. KL divergence ranges from 0 to infinity with 0 indicating that the probability distributions are a perfect match. With respect to this metric, Soffritto achieved the lowest mean KL divergence across bins in all cell lines. Notably, Soffritto’s predictions had a lower median KL divergence than the random forest model, with wider and tighter distributions around the median (Fig. 2C).

#### 3.1.3 Spearman correlation

The monotonic order of the S phase fractions in terms of replication probability is important to consider when evaluating a model’s predictions because it is more relevant biologically to capture whether a greater percentage of cells replicate a particular bin for one S phase fraction over another rather than predicting the exact replication probabilities. For this reason and for the interpretable nature of rank correlation, we included Spearman correlation as an additional metric in our analysis. For each bin, we compute a Spearman correlation between predicted and observed 16-fraction probability values. Here we found that Soffritto’s predictions highly correlate with observed values with median values ranging from 0.93 to 0.96 across the five cell lines. In addition, Soffritto’s predictions had a significantly higher mean correlation than all baseline models across all cell lines, except in HCT116 where the random forest model had the highest mean correlation (Fig. 2D).

#### 3.1.4 Kolmogorov–Smirnov and Wasserstein Distance

Another way of conceptualizing 16-fraction RT is by examining the cumulative replication fraction (Zhao *et al.* 2020). The cumulative replication fraction of a specific S phase fraction is defined as the cumulative sum of replication probabilities for all earlier fractions and the current fraction. Consequently, we obtain a vector length 16 for each bin that now represents a cumulative distribution rather than a probability distribution. In this space, one can infer the percentage of cells that replicated a genomic bin by a specific S phase fraction. We computed two metrics that can both be formulated as a measure of distance between two cumulative distribution functions (CDF): Kolmogorov–Smirnov (Massey 1951) and Wasserstein Distance (Kantorovich 1960). The Kolmogorov–Smirnov statistic is defined as the maximum absolute difference between empirical CDFs of two samples while the Wasserstein Distance implicitly computes the sum of the absolute differences between two CDFs. We chose both metrics because they capture both overall similarity between two CDFs and penalize the greatest error between two CDFs. We observed that Soffritto achieves a significantly lower mean Kolmogorov–Smirnov value than the baseline models in all cell lines, except for HCT116, for which Soffritto and random forest have performed similarly with respect to other metrics (Fig. 2E). As for Wasserstein Distance which captures a more global distance between CDFs, Soffritto achieves both a lower mean and median than all baselines in four out of the five cell lines, with particularly tighter distributions for the mouse cell lines (Fig. 2F). To quantify Soffritto’s overall performance, we ranked the models based on mean values for each of the five metrics and the five cell lines for a total of 25 rankings. We then computed the Borda count (Emerson 2013) for each ranking (0 points for the lowest-ranked model, 1 point for the second-lowest-ranked model, etc.) and summed the points for each model across all rankings. Soffritto was the highest ranked model with 122 out of a possible 125 points. Overall these results demonstrate that Soffritto’s predictions capture important RT characteristics in both probability space and CDF space.

### 3.2 Soffritto’s predicted RT profiles capture RT dynamics

**Figure 3.**
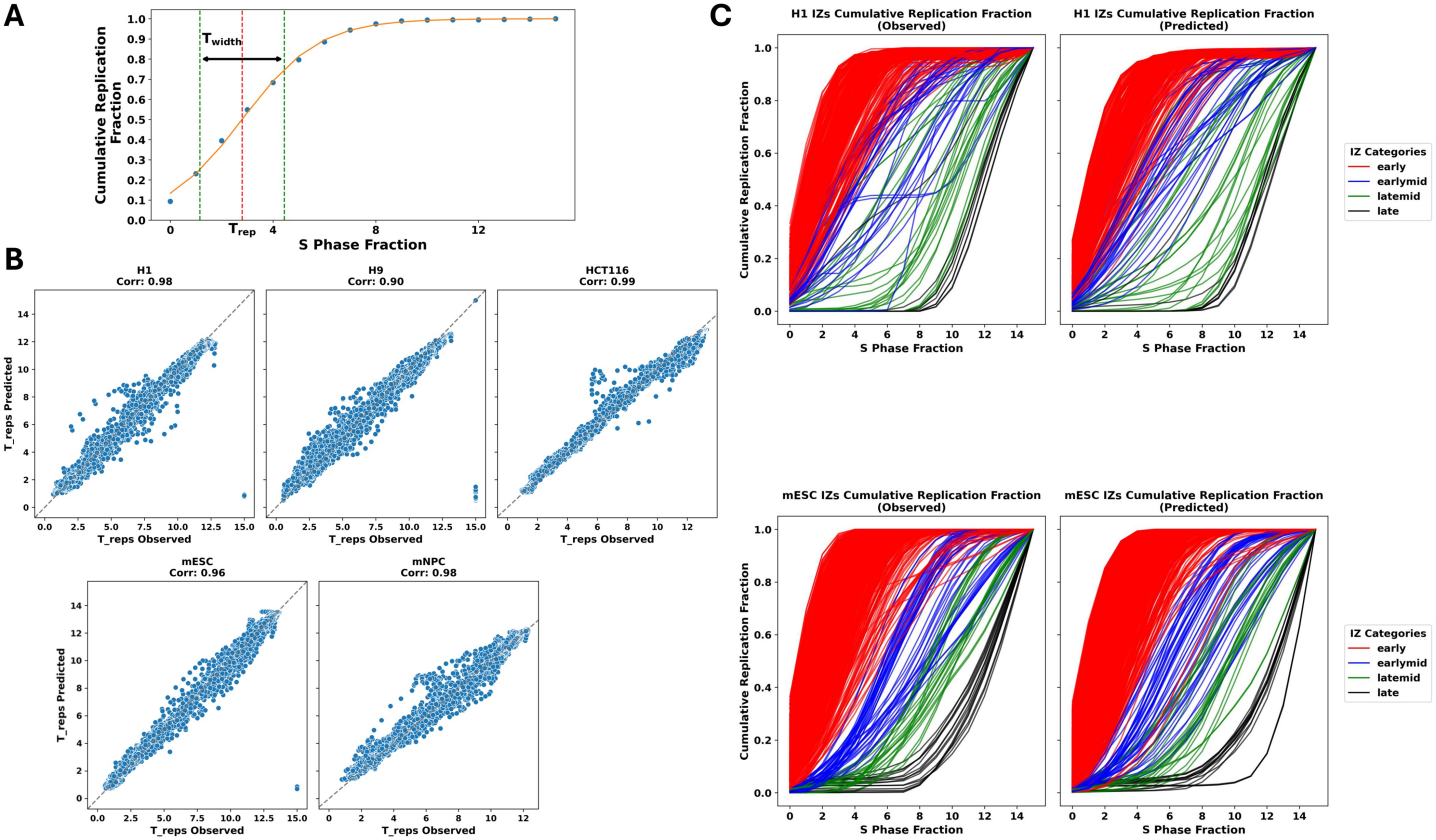
Dynamic RT features for observed versus predicted RT profiles. (A) Demonstration of *T*_rep_ and *T*_width_ calculation using a representative cumulative replication fraction distribution of a single bin. (B) Pearson correlation plots for observed versus predicted *T*_rep_ in all cell lines with each data point corresponding to a single bin of chromosome 9. The *y*-axis and *x*-axis correspond to predicted and observed *T*_rep_ values, respectively. (C) Observed versus predicted cumulative replication fraction curves grouped by temporal category in bins classified as IZs for H1 (top) and mESC (bottom). Each curve corresponds to a single bin of chromosome 9 that is categorized as IZ by Zhao *et al.* (2020).

Next, we aimed to examine whether Soffritto’s intra-cell line predictions captured high-resolution RT dynamics in the test chromosome. Two important features of RT dynamics that capture RT heterogeneity are *T*_rep_ and *T*_width_ (Zhao *et al.* 2020). *T*_rep_ is defined as the time point at which 50% of cells have finished replicating a genomic bin, while *T*_width_ is defined as the difference in time between when 75% of cells have finished replicating and 25% of cells have finished replicating. To compute *T*_rep_ and *T*_width_, a sigmoidal curve is fit to the cumulative replication fraction values for a particular bin (Fig. 3A). For our analysis, we express time in continuous values ranging from 0 (S1) to 15 (S16). We computed *T*_rep_ for both predicted and observed 16-fraction data for each bin in chromosome 9 for all five cell lines, and we found that Soffritto’s predicted *T*_rep_ values correlate highly with observed *T*_rep_ values, with a mean correlation of 0.96 (median 0.98) (Fig. 3B).

We then compared the replication dynamics of IZs for both observed and predicted 16-fraction RT to determine if Soffritto is capturing expected dynamics at these sites. IZs are defined as consecutive genomic bins that replicate earlier than flanking regions and at a roughly constant rate. They are thought to initiate replication as they are distributed throughout the genome and RT does not occur in a uniform manner genome-wide. In addition, IZs can be classified into four temporal categories based on their S phase fractions in which the highest read density was identified: early (S1–S3), early-mid (S4–S6), late-mid (S7–S9), and late (S10–S12) (Zhao *et al.* 2020). We obtained the genomic coordinates for IZs along with their corresponding temporal labels in H1 and mESC from (Zhao *et al.* 2020). We then computed the cumulative replication fraction for all bins located in IZs for both observed and predicted 16-fraction RT profiles on chromosome 9. We found that Soffritto’s predictions capture the increase in *T*_width_ as S phase progresses from early to mid, followed by a decrease in *T*_width_ from mid-S phase to late S phase in both H1 and mESC (Fig. 3C). These findings suggest that Soffritto’s predictions capture high-resolution temporal features that can be measured specifically in 16-fraction RT data.

### 3.3 Feature ablation analysis

**Figure 4.**
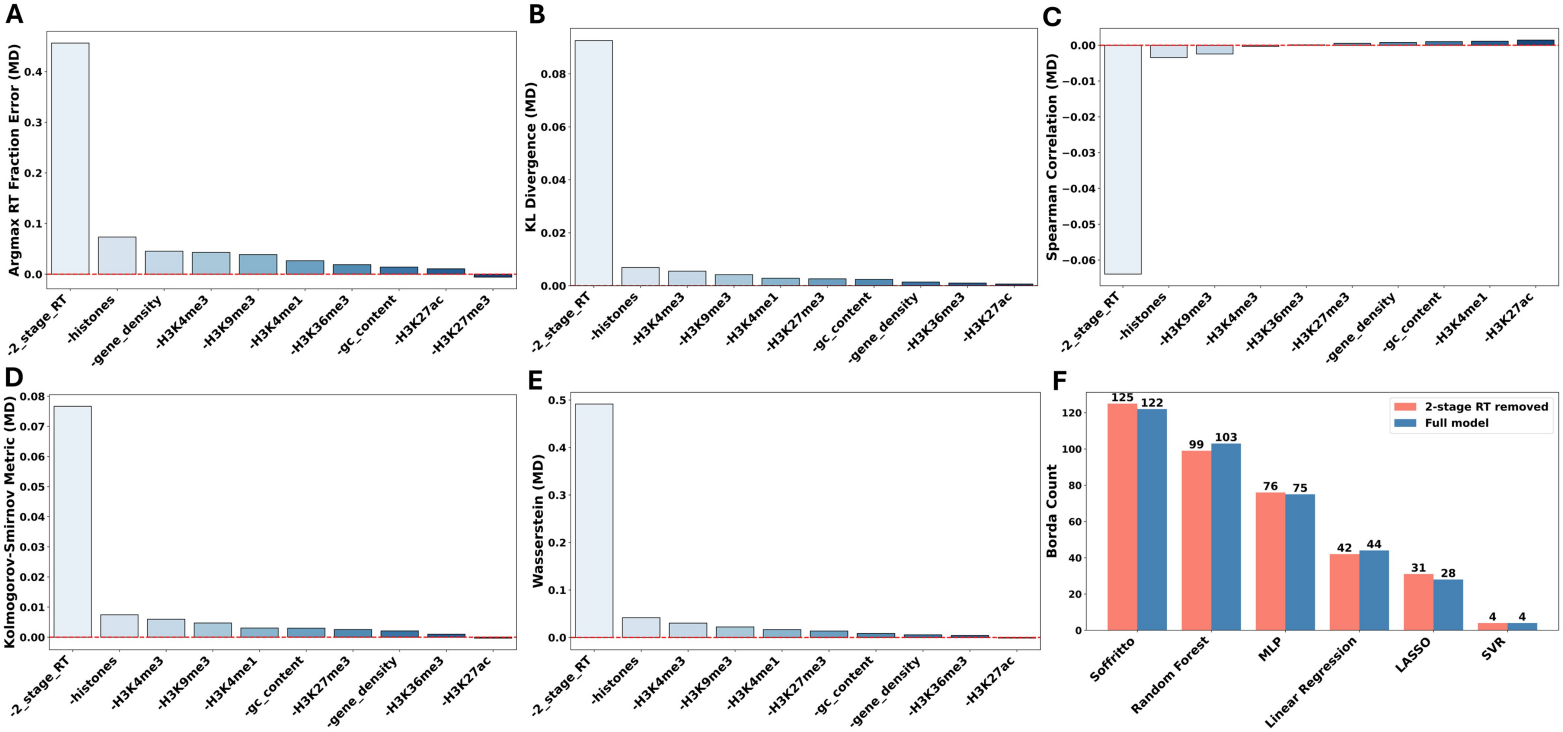
Ablation analysis for Soffritto’s predictions in mESC. (A) Each bar is labeled on the *x*-axis by the feature or set of features left out when re-training Soffritto. “-Histones” refers to all histone marks being left out. The *y*-axis measures the MD between the full feature model’s Argmax RT Fraction Errors and the corresponding ablated model’s Argmax RT Fraction Errors across all bins on chromosome 9. (B–E) Same plot but for KL divergence, Spearman correlation, Kolmogorov–Smirnov, and Wasserstein Distance, respectively. (F) Borda counts for Soffritto and the baseline models with the full set of features and with two-stage RT removed across all cell lines and metrics. The maximum possible Borda count for a model is 125 [five cell lines, five evaluation metrics, and a ranking score ranging 0 (worst) to 5 (best)].

We next performed a feature ablation analysis to assess each feature’s contribution to Soffritto’s performance. Using the same train-test splits within each cell line and the same hyperparameter configurations as the full model, Soffritto was re-trained by iteratively leaving out one feature at a time. In addition, the six histone marks were also collectively omitted to evaluate the overall effect of using histone marks as features. The ablated models’ predictions were then evaluated using the same five metrics. For each metric, the mean difference (MD) between the metric values for the full model and each ablated model was then computed. A higher positive (negative for Spearman correlation) MD for an ablated model thus indicates that the omitted feature has higher saliency. Across all metrics, two-stage RT was consistently the most salient feature in mESC (Fig. 4). This is expected because two-stage RT already captures the general 16-fraction RT signal (Zhao *et al.* 2020) and our predictions using one feature at a time already show this (Supplementary Fig. S2). While we found that removing histones collectively consistently led to the second largest drop in performance among the features, the histone marks varied in the amount of their individual effect. For mESC, H3K9me3 (constitutive heterochromatin) and H3K4me3 (active promoters) were consistently more salient than the other histone marks (Fig. 4). H3K27ac (active enhancers and promoters) and H3K36me3 (transcriptional elongation) omission had almost no effect on prediction in four out of the five metrics for mESC (Fig. 4B–E). Gene density was deemed more salient than the other predictors with respect to ARFE, but not for the other metrics (Fig. 4A). We also note that the MDs were quite small, particularly with respect to Spearman correlation, and that even omitting two-stage RT only lead to an average increase of 0.5 in ARFE. These small changes suggest combinatorial effects of these features in predicting 16-fraction RT that compensate for the ablation of a single feature or even a subset of features. When we repeated the same ablation analysis for mNPC, the highest saliency for two-stage RT and the second highest for all histone marks remained consistent (Supplementary Fig. S3). In terms of specific histone marks, different evaluation metrics ranked different marks highly including H3K9me3 and H3K4me3, similar to mESC, as well as H3K4me1 (active and poised enhancers) and H3K27me3 (polycomb-mediated repression).

We next visually inspected Soffritto’s predictions when either two-stage RT or histones, the two most salient features in mESC, were omitted. As an example, we examined the 16-fraction RT heatmaps of observed, full model predictions, histone mark omitted model predictions, and two-stage RT omitted model predictions in 10–20 Mb region of chromosome 9 (Supplementary Fig. S4). We observed a marginal increase in smoothing of the 16-fraction profiles in the predictions from the full model to the histone mark omitted model to the two-stage RT omitted, particularly in the early replicating 13-15 Mb region (Supplementary Fig. S4). The two-stage RT omitted model also failed to capture some narrow peaks in the observed heatmap, such as at the 12.5, 15.5, and 17.5 Mb loci, thereby demonstrating two-stage RT’s importance in capturing the informative features of 16-fraction RT data. Similar visual comparative analysis for the same region using mNPC data confirmed the observations from the mESC data (Supplementary Fig. S5). Interestingly, in addition to multiple regions going in the opposite direction, this analysis also highlighted one region (15 Mb) for which the predictions of two-stage RT omitted model were closer to measured high-resolution RT profiles compared to those from the full model. The local dip observed for this 15 Mb region from high-resolution RT measurements was not present in two-stage RT measurements likely explaining the smoothing effect when 2-stage RT data is used as a feature in our predictive models (Supplementary Fig. S5). Overall, these results suggest that while two-stage RT is the most predictive feature, incorporation of other features with or without two-stage RT measurements may allow detection of important RT patterns captured by high-resolution RT experiments.

We also re-ran the five baseline models on the intra-cell line train-test splits while omitting two-stage RT as a feature to examine any comparative differences in performance between these models and Soffritto. We found that Soffritto performed slightly better with respect to the baseline models under two-stage RT omission than when all features were used, earning the maximum Borda count of 125 across all metrics and cell lines (Fig. 4F). Random forest, the second highest-performing model, saw its Borda count drop slightly from 103 with the full model to 99 with two-stage RT omission. These results suggest that Soffritto predictions are better compared to other baseline models even in the absence of two-stage RT data.

### 3.4 Soffritto’s predictions generalize to unseen cell lines

**Figure 5.**
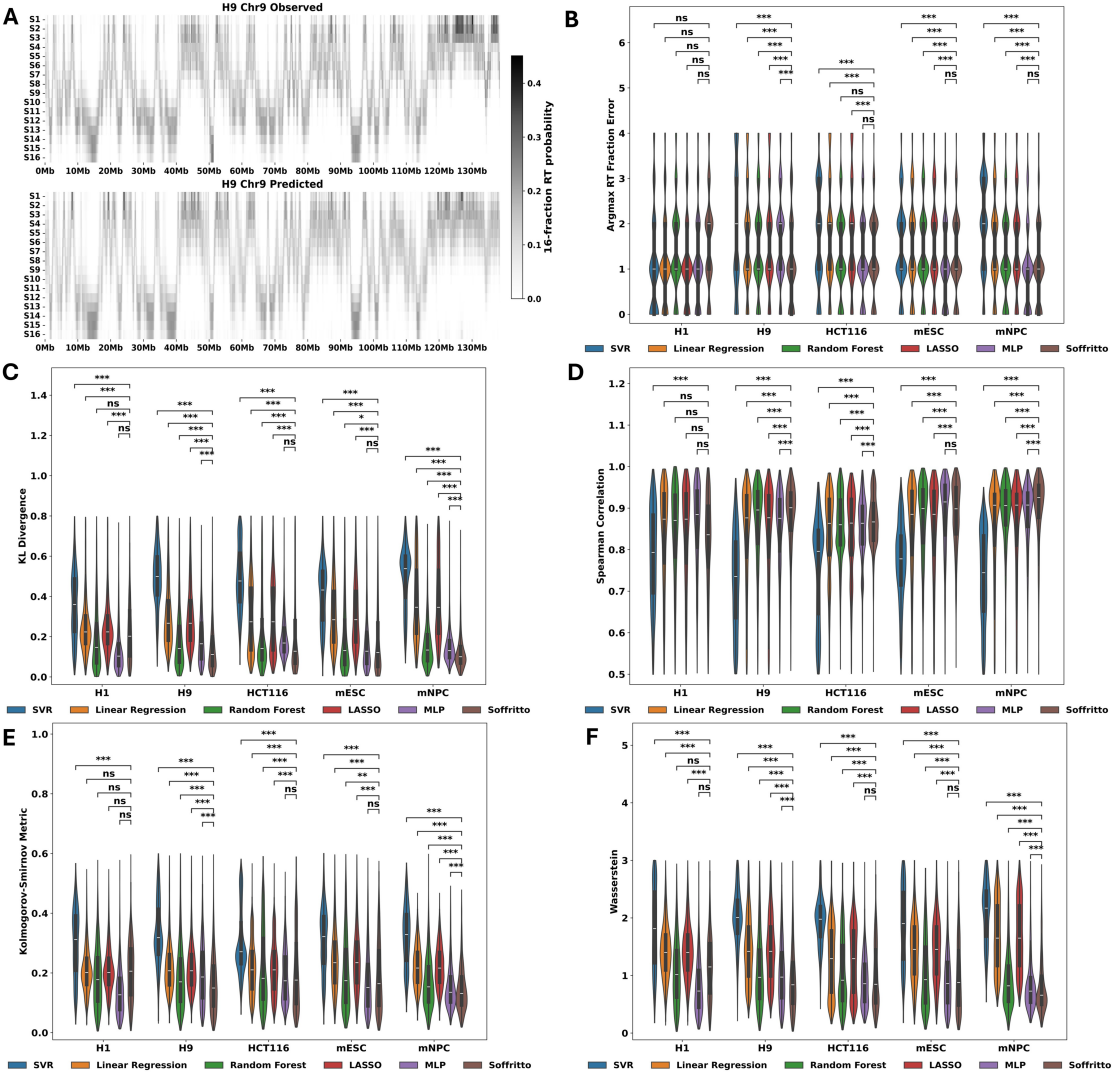
Leave-one-cell-line-out (cross-cell line) results. (A) Observed versus predicted RT heatmaps for left out H9. (B–F) Each panel corresponds to a different metric. Each cell line on the *x*-axis corresponds to the cell line left out when training the model. All *P*-values were computed and annotated with symbols as was described in Fig. 2.

We next wanted to test whether Soffritto could predict 16-fraction RT in an unseen cell line when trained on data from multiple other cell lines. To do this we implemented a leave-one-cell-line-out strategy by iteratively leaving one cell line out while training on the other four cell lines (Section 2). We omitted chromosome 9 from the training data to ensure an unseen chromosome in the test set to account for potential model memorization from the sequence-based features (with the caveat that there is no synteny between human chr9 and mouse chr9). We observed that Soffritto’s predicted RT captured the general RT trends of chromosome 9 to a good extent (Fig. 5A; median Spearman correlation of 0.9). For a subset of regions (e.g. the last 20 Mb of chromosome 9), the predicted RT showed a wider distribution across adjacent fractions compared to observed profiles (Fig. 5A).

We also trained the same five baseline models (SVR, linear regression, random forest, LASSO, and MLP) as in the intra-cell line evaluation on this cross-cell line setting. We evaluated Soffritto using the same five metrics as the intra-cell line analysis. Soffritto achieved a significantly lower mean ARFE than four out of the five baseline models for three of the five left out cell lines, being on average 1.4 S phase fractions off (Fig. 5B). Figure 5C shows that Soffritto has a significantly lower mean KL divergence for H9 and mNPC with comparable performance between Soffritto and MLP in mESC (Fig. 5C). Soffritto also achieved a mean Spearman correlation of 0.85 across all left out cell lines, higher than the baseline models for all cell lines. Within cell lines, Spearman correlation indicates that Soffritto has lower performance than most of the baseline models in H1 (Fig. 5D). With respect to the cumulative replication fraction metrics, Soffritto’s predictions do slightly better according to Wasserstein Distance, having a significantly higher mean distance than LASSO and Linear Regression in H1 (Fig. 5E and F). In terms of Soffritto’s overall performance, we ranked the models by computing the Borda count for each ranking and summing the points for each model across all rankings. For each cell line there are 25 possible points, and we found that Soffritto was the highest ranked model for two out of five (H9 and mNPC) and the second highest ranked in another two cell lines (HCT116 and mESC). Across all cell lines combined, Soffritto was the second highest-performing model, gathering 102 out of 125 possible points and MLP, the highest-performing model, got 110 points. Overall, these results demonstrate that both Soffritto and MLP models are able to predict 16-fraction RT patterns in unseen cell lines with reasonable accuracy. Robust training and evaluation of such cross-cell line models will require a larger set of high-resolution RT experiments that cover distinct cell lines/types.

## 4 Discussion

We introduced Soffritto, an LSTM-based model that predicts 16-fraction RT data using two-fraction RT, six histone modifications, GC content, and gene density as input. As the first predictive model of 16-fraction RT, Soffritto outperforms non-recurrent baseline models in intra-cell line and cross-cell line experiments in five human and mouse cell lines. The five metrics we used for evaluation capture different aspects of the 16-fraction RT signal, such as the fraction with the highest probability and the cumulative replication fraction distribution, that are biologically meaningful. We also showed that Soffritto accurately captures patterns of dynamic RT features defined in experimental 16-fraction RT data. Soffritto’s embeddings capture broad RT trends across bins as well as centromeres, which are known for having unstable replication profiles.

Although Soffritto’s predictions for unseen cell types had reasonable accuracy, an increase in the availability of high-resolution (16 or similar number of fractions) RT data could provide substantial benefit to its generalizability. In this work, we used a small set of features to make Soffritto applicable to a broader set of cell types; however, our model can certainly be expanded to include more features either from DNA sequence or cell-type-specific functional genomics measurements. We also note that while Soffritto was the best-performing model overall, random forest had comparable performance for some of the cell lines and metrics in the intra-cell line evaluation while the MLP model performed slightly better overall in the leave-one-cell-line out approach, suggesting that the input features for each bin have high predictive power even when considered in isolation from neighboring bins. When we quantified autocorrelation to understand this better, we observed 16-fraction RT exhibits high autocorrelation with neighboring bins as expected. This was also the case for two-stage RT, which largely captured the same autocorrelation pattern across different bin lags (Supplementary Fig. S6). Consequently, using two-stage RT as a feature allows non-sequential models like random forest and standard MLPs to perform well in these 16-fraction RT prediction tasks. Future improvements for Soffritto may include utilization of input features at higher resolution and/or exploring more sophisticated deep learning architectures that may exploit longer-range correlations of input features or physical proximity of linearly distant bins in 3D space.

## Supplementary Material

btaf231_Supplementary_Data

## Data Availability

All 16-fraction RT data was downloaded from the Gene Expression Omnibus at accession GSE137764 (Zhao, Sasaki and Gilbert 2020). The code developed in this work and the processed data used in our predictive models are made available at: https://github.com/ay-lab/Soffritto.
