# Supplementary material for: Soffritto: a deep learning model for predicting high-resolution replication timing
Source: Bioinformatics. 2025 Jul 15;41(Suppl 1):i580–9. doi: 10.1093/bioinformatics/btaf231 (PMC12261407; doi:10.1093/bioinformatics/btaf231)
Supplement: btaf231_Supplementary_Data [file btaf231_supplementary_data.zip › btaf231_Supplementary_Data/Bolzan.347.Soffritto_alt_text.pdf]

Figure 1 Alt text*: Soffritto model diagram of its input, LSTM module, prediction module, and output.*

Figure 2 Alt text*: Six panels labeled A to F. A shows an observed and predicted 16-fraction heatmap stacked vertically. Panels B through F each correspond to a metric, showing a series of violin plots comparing Soffritto to the baseline models for each cell line. The violin plots are annotated with p-values.*

Figure 3 Alt text*: Panels labeled A to C. A is a Cumulative Replication Fraction plot with S phase Fraction on the x-axis and cumulative replication fraction on the y-axis. B is a series of five correlation plots, one for each cell line. C contains two plots, each showing cumulative replication fraction subplots for observed and predicted RT profiles in IZs.*

Figure 4 Alt text*: Panels labeled A to F. Panels A to E each show a bar plot, with each bar corresponding to an ablated feature. Panel F is a bar plot displaying Borda counts, with bars grouped by model and labeled as either full model or 2-stage RT removed model.*

Figure 5 Alt text*: Six panels labeled A to F. A shows an observed and predicted 16-fraction heatmap stacked vertically. Panels B through F each correspond to a metric, showing a series of violin plots comparing Soffritto to the baseline models for each cell line left out. The violin plots are annotated with p-values.*

Supplementary Table 1 Alt text*: Table of accession numbers indicating data source for each epigenetic feature for each cell line.*

Supplementary Table 2 Alt text*: Table of optimal hyperparameter values for each cell line and each hyperparameter. The hyperparameters shown are the learning rate, hidden dimension, batch size, number of LSTM layers, and L2 weight decay.*

Supplementary Table 3 Alt text*: Table of optimal hyperparameter values for each cell line and each hyperparameter. The hyperparameters shown are the learning rate, hidden dimension, batch size, number of LSTM layers, and L2 weight decay.*

Supplementary Figure 1*: Panel A represents intra-cell line train, validation, and test splits as a rectangle for each cell line, highlighting chromosome 6 and chromosome 9 as the validation and test set respectively. Panel B represents leave-one-cell-line-out splits as a rectangle with the rows corresponding to chromosomes and the columns corresponding to cell lines, highlighting chromosome 6 as the validation set in cell lines used for training and chromosome 9 as the test set in the left-out cell line.*

Supplementary Figure 2*: Heatmap with rows representing features and columns representing cell lines, where each value is the mean argmax RT fraction error for individual feature predictions.*

Supplementary Figure 3*: Five panel figure where panels A, B, C, D, and E correspond to the metrics argmax RT fraction error, KL divergence, Spearman correlation, Kolmogorov-Smirnov, and Wasserstein respectively. Each panel is a series of bar plots where the y-axis is the Mean Difference, and the x-axis is labeled according to the feature left out during training with a "-" sign preceding the feature's name.*

Supplementary Figure 4*: Four heatmaps stacked vertically where each entry is the 16-fraction RT probability for a 50kb bin and an S phase fraction. The y-axis corresponds to the S phase fraction, labeled S1 to S16 going from top to bottom, and the x-axis corresponds to the 50kb bins.*

Supplementary Figure 5*: Four heatmaps stacked vertically where each entry is the 16-fraction RT probability for a 50kb bin and an S phase fraction. The y-axis corresponds to the S phase fraction, labeled S1 to S16 going from top to bottom, and the x-axis corresponds to the 50kb bins.*

Supplementary Figure 6*: Six panel figure arranged with two panels per row. Each row corresponds to a chromosome. The first column of figures corresponds to 2-stage RT and the second column corresponds to 16-fraction RT. Each panel plots autocorrelation as a function of lag. Lag values range from 0 to the number of 50kb bins in the respective chromosome.*
