# Supplementary material for: Soffritto: a deep learning model for predicting high-resolution replication timing
Source: Bioinformatics. 2025 Jul 15;41(Suppl 1):i580–9. doi: 10.1093/bioinformatics/btaf231 (PMC12261407; doi:10.1093/bioinformatics/btaf231)
Supplement: btaf231_Supplementary_Data [file btaf231_supplementary_data.zip › btaf231_Supplementary_Data/Bolzan.347.sup.1.pdf]

SUPPLEMENTARY INFORMATION for:

Soffritto: a deep-learning model for predicting high-resolution replication timing

SUPPLEMENTARY TABLES

Supplementary Table 1: Data sources of non-sequence based features. Histone ChIP-seq data was downloaded from ENCODE with the exception of mNPC. The 2-stage Repli-Seq data was downloaded from the 4DN Portal.

	**H1**	**H9**	**HCT116**	**mESC**	**mNPC**
**H3K27ac**	ENCFF423TVA	ENCFF988WEQ	ENCFF169MCH	ENCFF163SBS	GSE96107
**H3K27me3**	ENCFF380KPI	ENCFF077DRH	ENCFF717ZKL	ENCFF182FTP	GSE96107
**H3K36me3**	ENCFF370UNF	ENCFF565AYJ	ENCFF024LGD	ENCFF097KTK	GSE96107
**H3K4me1**	ENCFF396RXV	ENCFF142KLG	ENCFF337BPL	ENCFF280RYX	GSE96107
**H3K4me3**	ENCFF730VVX	ENCFF462TXF	ENCFF649ZLF	ENCFF719IZR	GSE96107
**H3K9me3**	ENCFF350QBK	ENCFF891UKM	ENCFF989AAM	ENCFF519ZAT	GSE96107
**2-stage Repli-Seq**	4DNESEJCRVNR	4DNESDB2JQ5S	4DNESXPNEE4Q	4DNES3BNI8G3	4DNESSLDL428

Supplementary Table 2: Intra-cell line optimal hyperparameter configurations for Soffritto for each cell line.

	**Learning Rate**	**Hidden Dimension**	**Batch size**	**LSTM layers**	**L2 Weight Decay**
**H1**	0.001	64	64	4	0.0001
**H9**	0.001	128	32	4	1e-05
**HCT116**	0.001	64	64	4	0.001
**mESC**	0.0001	32	64	4	0.0
**mNPC**	0.001	16	64	4	0.001

Supplementary Table 3: Leave one cell line out optimal hyperparameter configurations for Soffritto for each cell line left out.

	Learning Rate	Hidden Dimension	Batch size	LSTM layers	L2 Weight Decay
**H1 out**	0.0001	32	64	3	0.0001
**H9 out**	0.0001	64	64	4	0.001
**HCT116 out**	0.0001	32	64	3	0.0001
**mESC out**	0.0001	32	32	3	0.0
**mNPC out**	0.0001	64	64	3	0.0001

SUPPLEMENTARY FIGURES

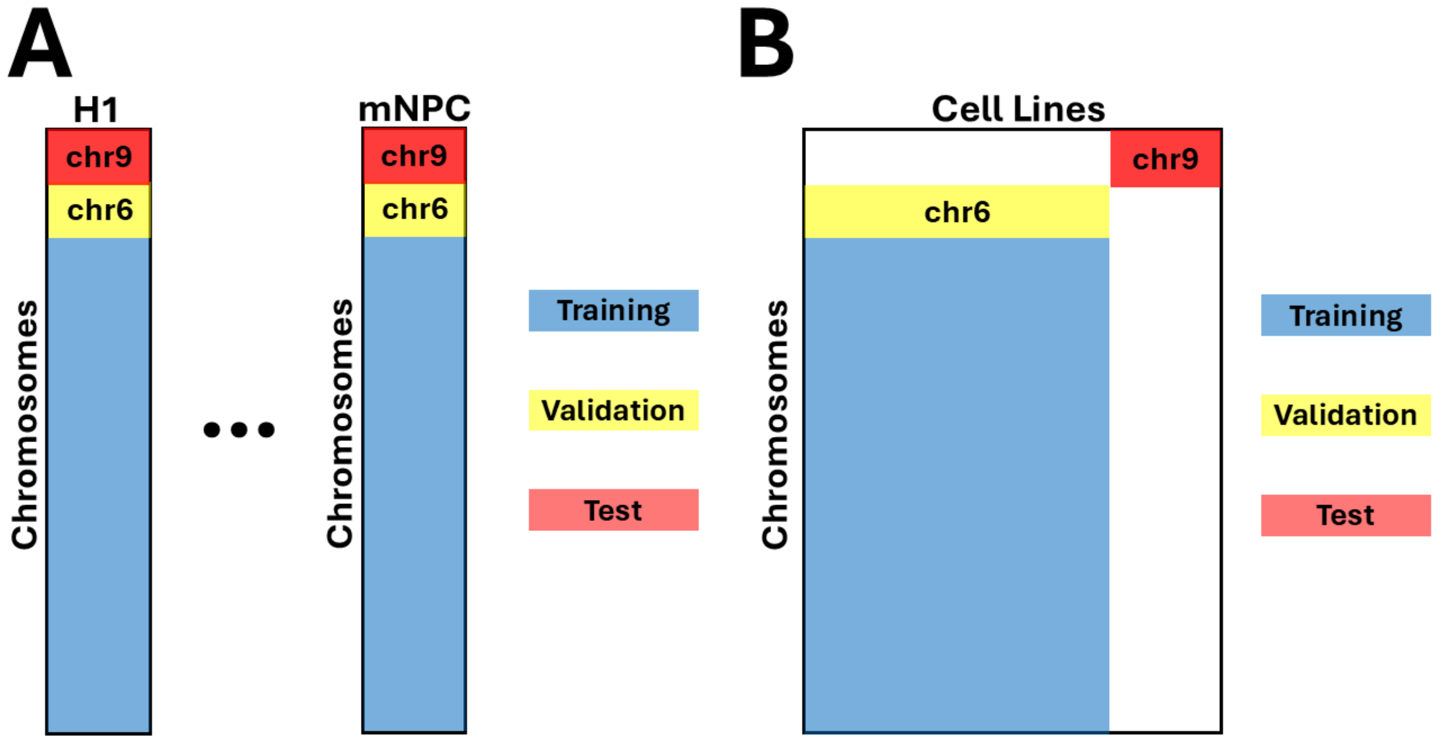

Supplementary Figure 1: Train-test splits for intra-cell line (**A**) and leave-one-cell-line-out (**B**) schemes.

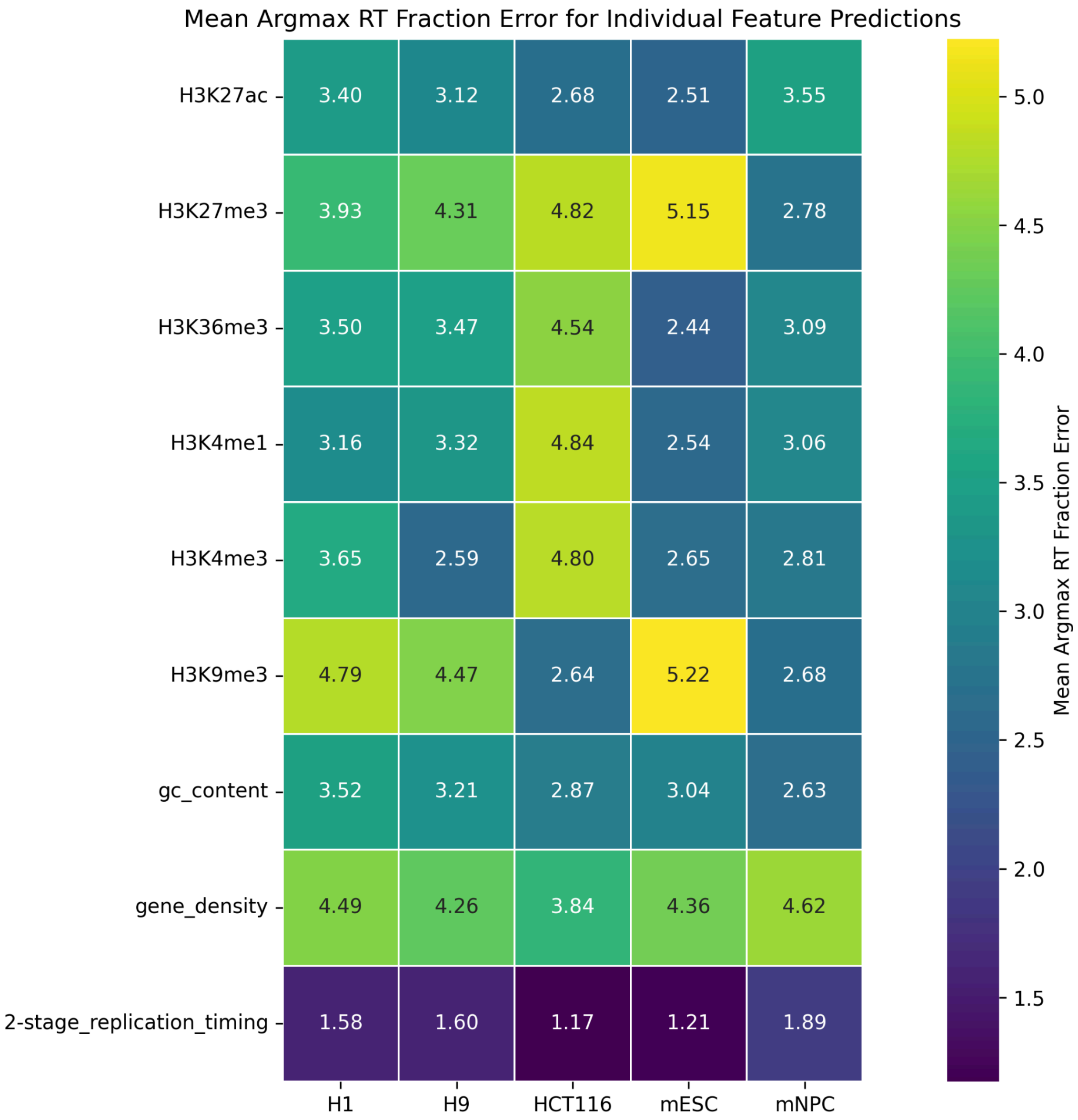

Supplementary Figure 2: Mean Argmax RT Fraction Error when predicting from individual features for chromosome 9 for each cell line.The values for each feature were sorted from lowest to highest and then partitioned into 16 non-overlapping intervals. Each interval was assigned a fraction S1-S16 based on ascending or descending order. Each genomic bin was then assigned a fraction based on the interval its feature value fell into. The mean absolute error (chromosome-wise) of these predictions was then computed with respect to the Argmax RT fractions of the observed data for both ascending and descending sorting. The min Argmax RT Fraction Error over both sortings is reported here.

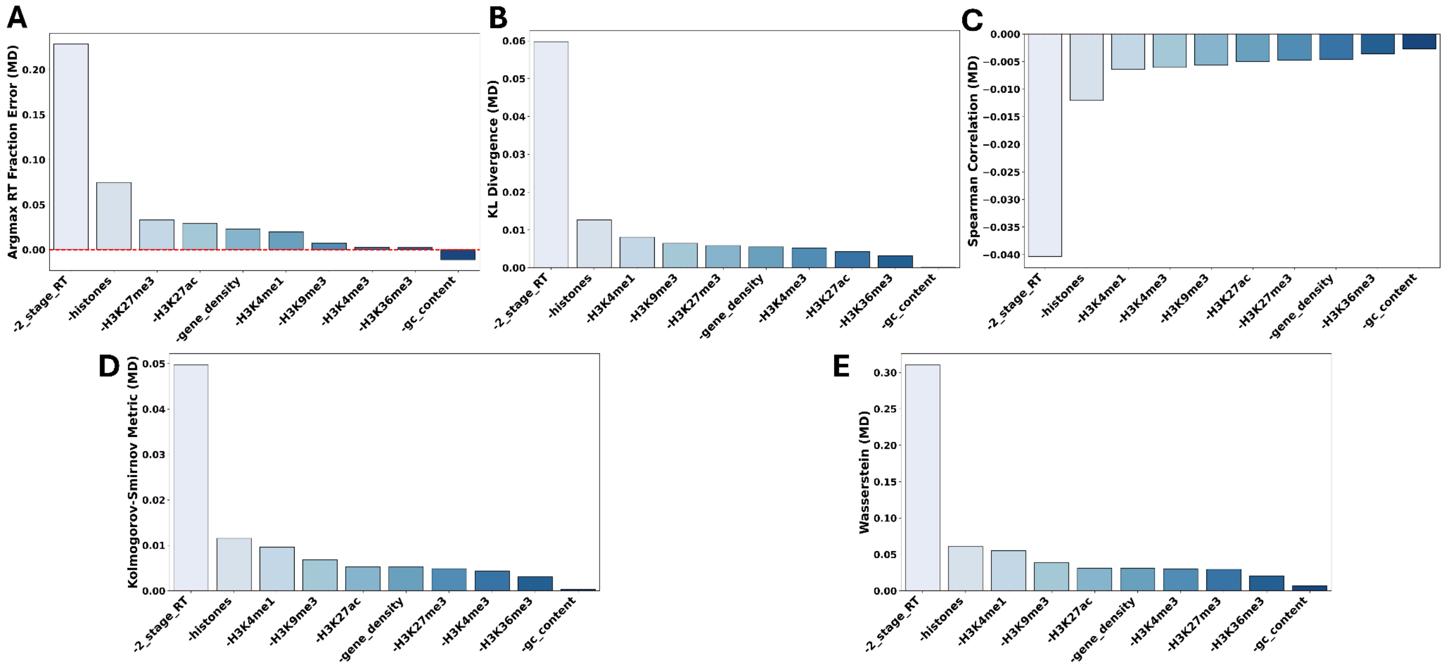

Supplementary Figure 3: mNPC ablation bar plots. Mean Difference (MD) between full model metric values and ablated model metric values across all bins on chromosome 9. Each bar is labeled according to the feature(s) that was left out. “-Histones” corresponds to all six histone marks.

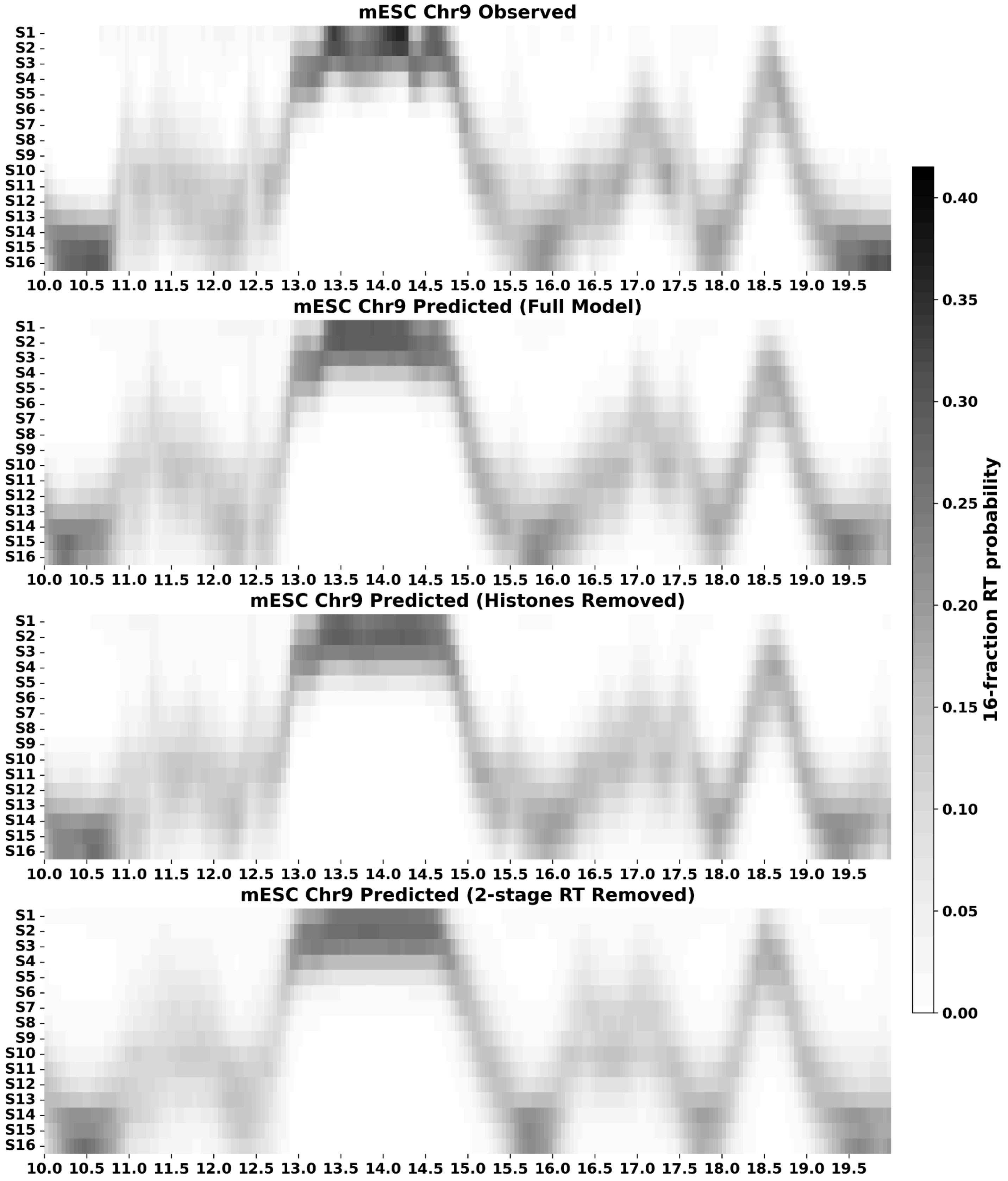

Supplementary Figure 4: 16-fraction RT heatmaps for full and ablated models for mESC chr9:10Mb-20Mb. Going from top to bottom: Observed, full model predictions, model with histones removed predictions, and model with 2-stage RT removed predictions. The x-axis labels are in units of Mb.

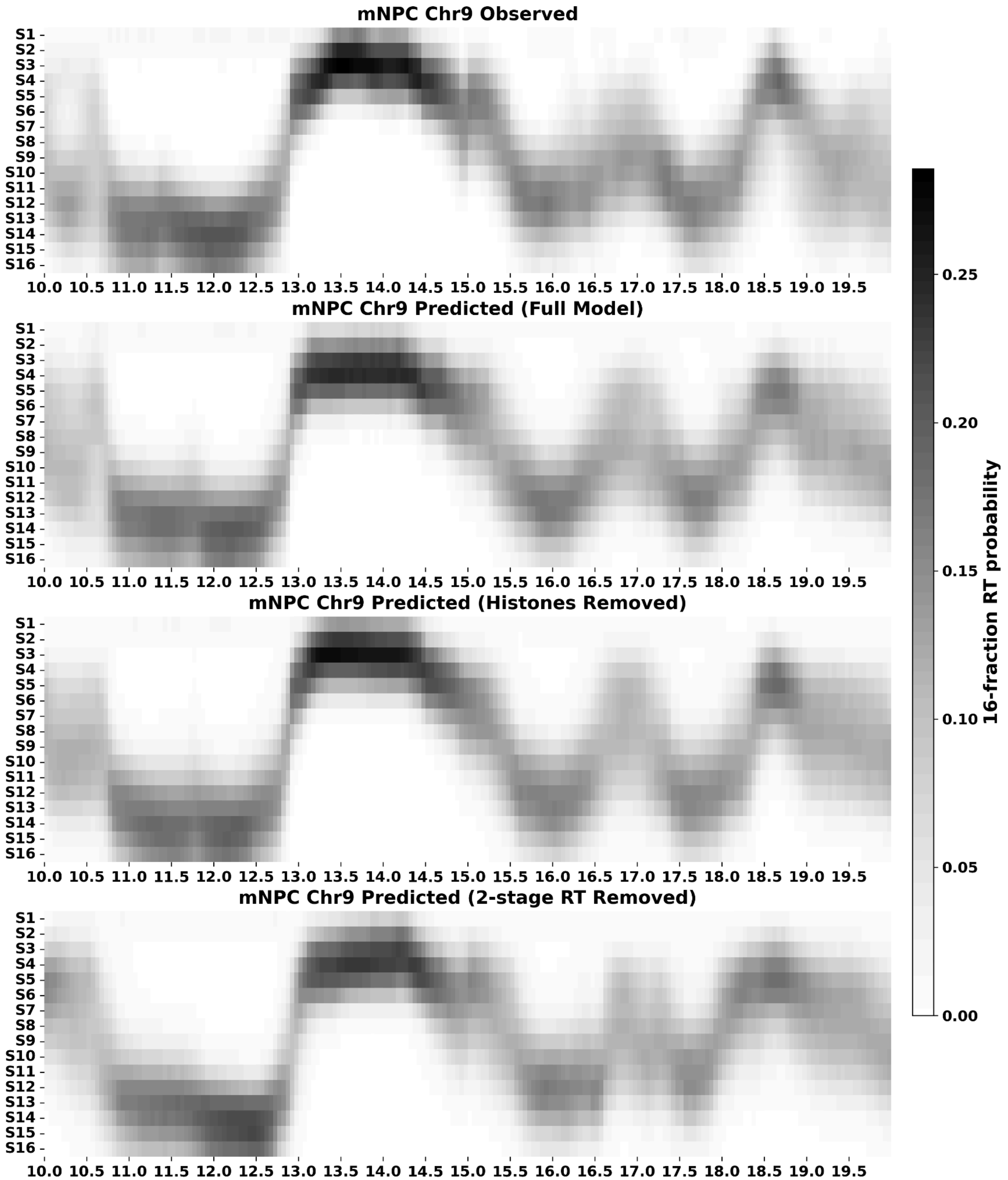

Supplementary Figure 5: 16-fraction RT heatmaps for full and ablated models for mNPC chr9:10Mb-20Mb. Going from top to bottom: Observed, full model predictions, model with histones removed predictions, and model with 2-stage RT removed predictions. The x-axis labels are in units of Mb.

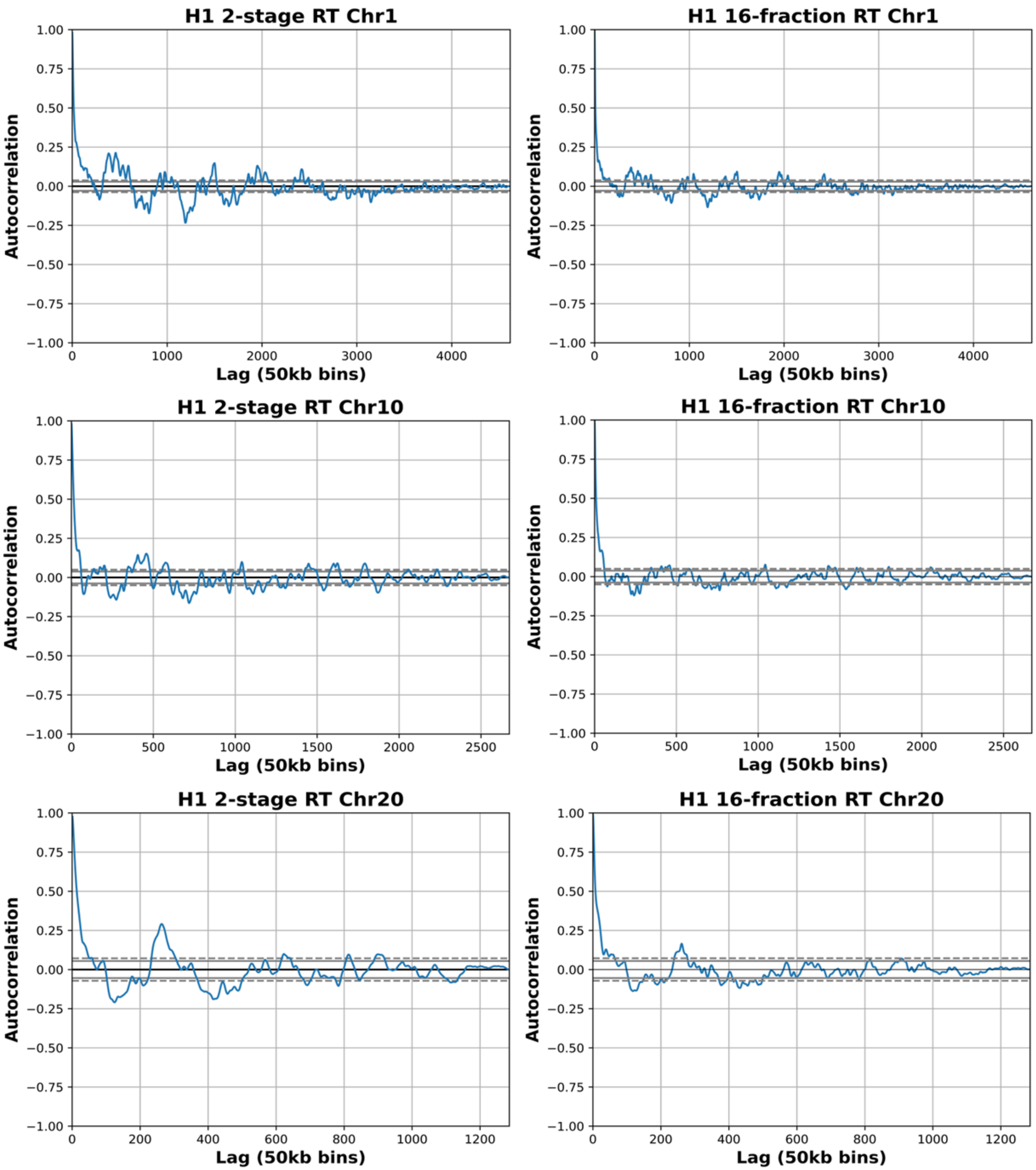

Supplementary Figure 6: Autocorrelation plots for 2-stage and 16-fraction RT data for H1. Each row corresponds to chromosomes 1, 10, and 20 respectively. The solid and dashed horizontal lines represent 95% and 99% confidence respectively for autocorrelation under the assumption that the series data is Gaussian noise. Values above or below the confidence bands indicate significant autocorrelation.
